# Inhibition of exportin-1 function results in rapid cell cycle-associated DNA damage in cancer cells

**DOI:** 10.18632/oncotarget.17063

**Published:** 2017-04-12

**Authors:** Russell T. Burke, Joshua M. Marcus, James D. Orth

**Affiliations:** ^1^ Department of Molecular, Cellular and Developmental Biology, University of Colorado Boulder, Boulder, CO, USA; ^2^ Current/Present address: Cell, Molecular and Developmental Biology, Graduate Biomedical Sciences, University of Alabama Birmingham, Birmingham, AL, USA

**Keywords:** XPO1, DNA damage, cell cycle, selinexor, therapeutic combinations

## Abstract

Selective inhibitors of nuclear export (SINE) are small molecules in development as anti-cancer agents. The first-in-class SINE, selinexor, is in clinical trials for blood and solid cancers. Selinexor forms a covalent bond with exportin-1 at cysteine-528, and blocks its ability to export cargos. Previous work has shown strong cell cycle effects and drug-induced cell death across many different cancer-derived cell lines. Here, we report strong cell cycle-associated DNA double-stranded break formation upon the treatment of cancer cells with SINE. In multiple cell models, selinexor treatment results in the formation of clustered DNA damage foci in 30-40% of cells within 8 hours that is dependent upon cysteine-528. DNA damage strongly correlates with G1/S-phase and decreased DNA replication. Live cell microscopy reveals an association between DNA damage and cell fate. Cells that form damage in G1-phase more often die or arrest, while those damaged in S/G2-phase frequently progress to cell division. Up to half of all treated cells form damage foci, and most cells that die after being damaged, were damaged in G1-phase. By comparison, non-transformed cell lines show strong cell cycle effects but little DNA damage and less death than cancer cells. Significant drug combination effects occur when selinexor is paired with different classes of agents that either cause DNA damage or that diminish DNA damage repair. These data present a novel effect of exportin-1 inhibition and provide a strong rationale for multiple combination treatments of selinexor with agents that are currently in use for the treatment of different solid cancers.

## INTRODUCTION

Selective inhibitors of nuclear export (SINE) are a first-in-class family of compounds with potential clinical application in different disease states, including inflammation, autoimmunity, ALS and cancers [1–6]. SINE action works through direct, slowly-reversible covalent binding to the karyopherin exportin-1 (XPO1/CRM1) at cysteine-528 located in the cargo-binding cleft [7–9]. SINE binding to XPO1 prevents access of the cargo nuclear export sequence (NES) to the binding pocket, resulting in the subsequent nuclear accumulation and retention of cargo proteins [9].

Numerous studies use SINE to probe the anti-cancer potential of inhibiting XPO1 function [8, 10–12]. Within hours of SINE treatment, cargo sequestration, cell cycle arrest and progression defects, and activation of apoptosis are observed [10, 13]. Flow cytometry experiments in several studies report G1-phase accumulation and a rapid loss of the S-phase population after inhibition of XPO1 [13]. It is unclear from these studies what the fates of the cells accumulating in G1/S-phase are: progression and division, arrest, or death. Cell cycle effects and apoptosis occur in many different cancer-derived cell lines and xenograft models with SINE, indicating potential broad efficacy of XPO1 as an anti-cancer target [8, 13, 14]. Single cell longitudinal tracking using the fluorescent ubiquitin cell cycle indicator (FUCCI) system in HT-1080 fibrosarcoma cells shows that after acute treatment with selinexor (KPT-330) many cells treated in G1-phase exhibit G1-phase cell cycle arrest associated with cell death [10]. Some cells treated in G1-phase progress to cell division. These cells, and those treated in early S-phase, often show a protracted S-phase progression that is at least 2-fold longer than untreated cells, and some of these cells die in S-phase [10]. Cell stresses that may account for arrest and cell death in G1-phase and S-phase associated phenotypes –or– that in turn may be caused by abnormal S-phase progression, are unclear after selinexor treatment.

DNA damage can cause cell cycle arrest and death [15, 16]. Likewise, abnormal DNA synthesis can result in DNA double-stranded breaks and S-phase arrest or progression defects [17–19]. Our and other's observations of G1-phase arrest and S-phase effects after SINE treatment prompted us to examine if there is a relationship between inhibition of XPO1 with SINE and DNA double-stranded damage.

## RESULTS

### DNA damage after SINE treatment depends on XPO1 Cysteine-528

**Figure 1:**
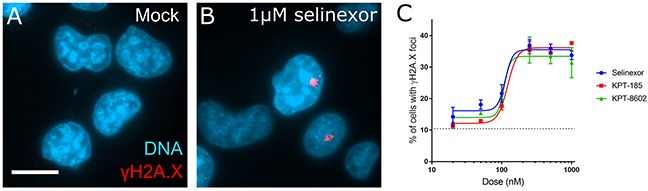
DNA damage foci formation is dose-dependent in response to SINE treatment **(A, B)** HT-1080 cells were treated for 8 hours with DMSO (mock) or 1μM selinexor. Cells were fixed and stained for the phosphorylated histone variant γH2A.X (red) and DNA (blue). Prominent damage foci are present in selinexor treated cells. **(C)** Multiple XPO1 inhibitors show dose-dependent foci formation. Points represent the mean percent of cells at each dose with γH2A.X foci. The dotted line is the mock treated population. Error bars are the SEM from three replicate experiments, at least 100 cells scored in each. Note: The KPT-185 enantiomer KPT-301 does not cause foci formation (not shown). Scale bar in A = 10μm for all panels.

Cell-based effects after XPO1 inhibition by SINE begin within hours of treatment, including the nuclear sequestration of cargos, cell cycle effects, and cell death [6, 10, 12]. The cell cycle effects are complex, including arrest and progression defects as characterized by flow cytometry and time-lapse microscopy with longitudinal tracking of cells [10, 13]. After 8 hours of acute treatment with SINE, we observe cell cycle progression defects, including in S-phase cells, before cell death occurs [10]. Cell cycle effects and cell death often associate with DNA damage. We asked whether DNA damage occurs after treatment with SINE. Fixed cell analyses of HT-1080 cells after 8 hours of SINE indicate dose-dependent double-stranded DNA damage in 35-40% of cells via immunostaining for the phosphorylated serine-139 histone variant H2A.X (γH2A.X) (Figure 1). Three different SINE compounds – selinexor (KPT-330), KPT-8602, and KPT-185 – each cause foci formation to the same extent (Figure 1C). The tool compound KPT-301, the inactive trans isomer of KPT-185, at 1μM shows no increase in the number of cells with DNA damage foci over mock treated cells (0.05% DMSO) (data not shown).

**Figure 2:**
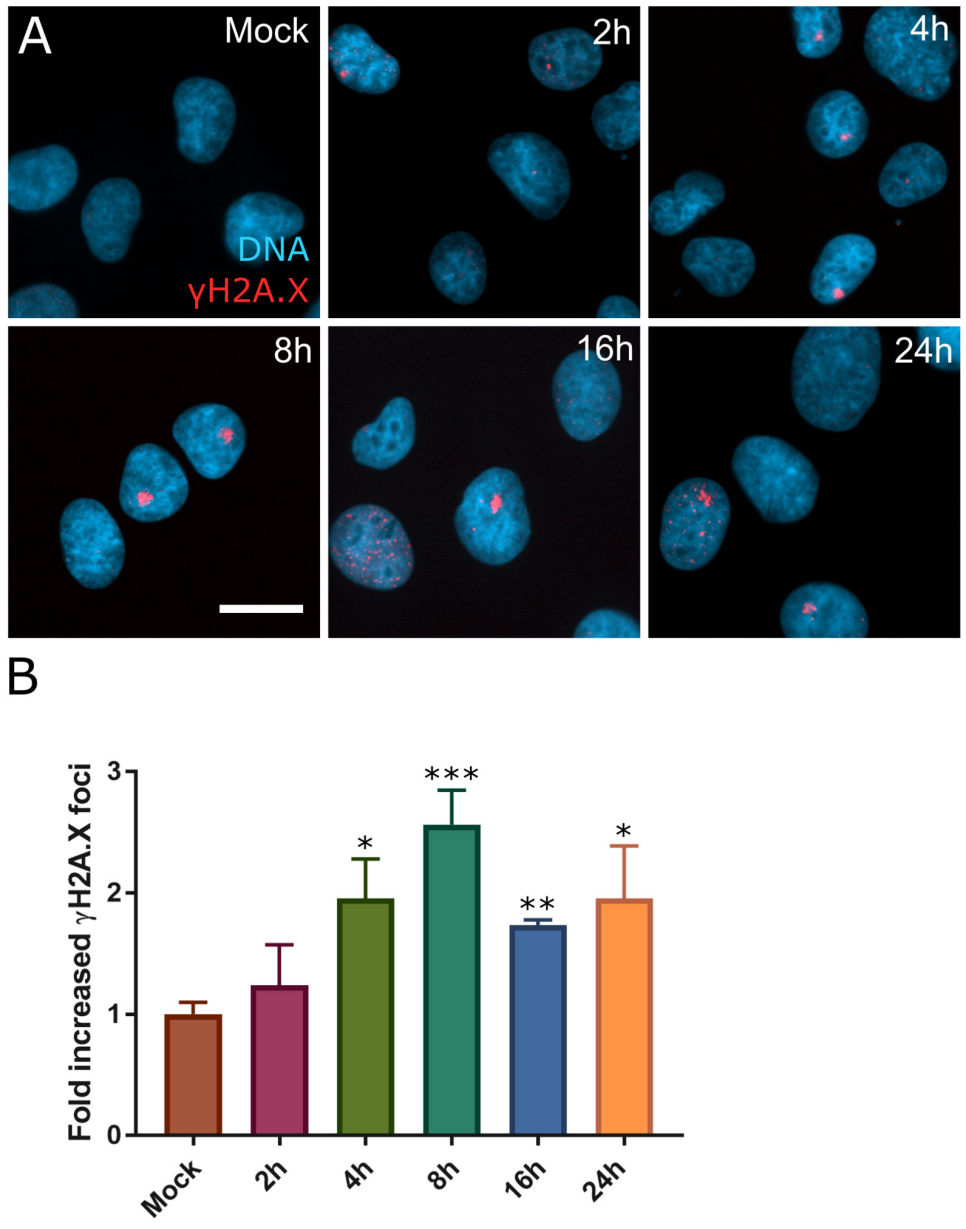
DNA damage foci form rapidly after SINE treatment **(A)** HT-1080 cells were treated with DMSO (mock) or 1μM selinexor for 2, 4, 8, 16, or 24 hours (h). Cells were fixed and stained for γH2A.X (red) and DNA (blue). **(B)** Mean fold increase in cells with γH2A.X foci over mock treated cells for each time point was scored. Error bars are the SEM from three replicate experiments, at least 100 cells scored in each. A Student's t-test was performed comparing time points to mock treated. *** is p<0.001, ** is p<0.01 and * is p<0.05. Scale bar = 10μm for all panels.

Many previous studies use 1μM to study SINE response and it can be achieved *in vivo* [8, 11, 20]. Unless noted otherwise, selinexor is used. In HT-1080, foci formation after selinexor treatment peaks after 8 hours and remains elevated over mock at 24 hours (Figure 2). In addition to HT-1080 cells, MCF7 breast carcinoma, U2OS osteosarcoma, HCT116 colon carcinoma, HeLa cervical carcinoma, and PANC-1 pancreatic carcinoma, cells show DNA damage foci after treatment with selinexor (Supplementary Figure 1A-1J). Interestingly, two proliferative, non-transformed human cell lines, telomerase immortalized retinal pigment epithelial (RPE1) and mesenchymal stem cells (MSC), show no strong increase in γH2A.X foci staining after treatment with 1μM selinexor (Supplementary Figure 1K-1P).

**Figure 3:**
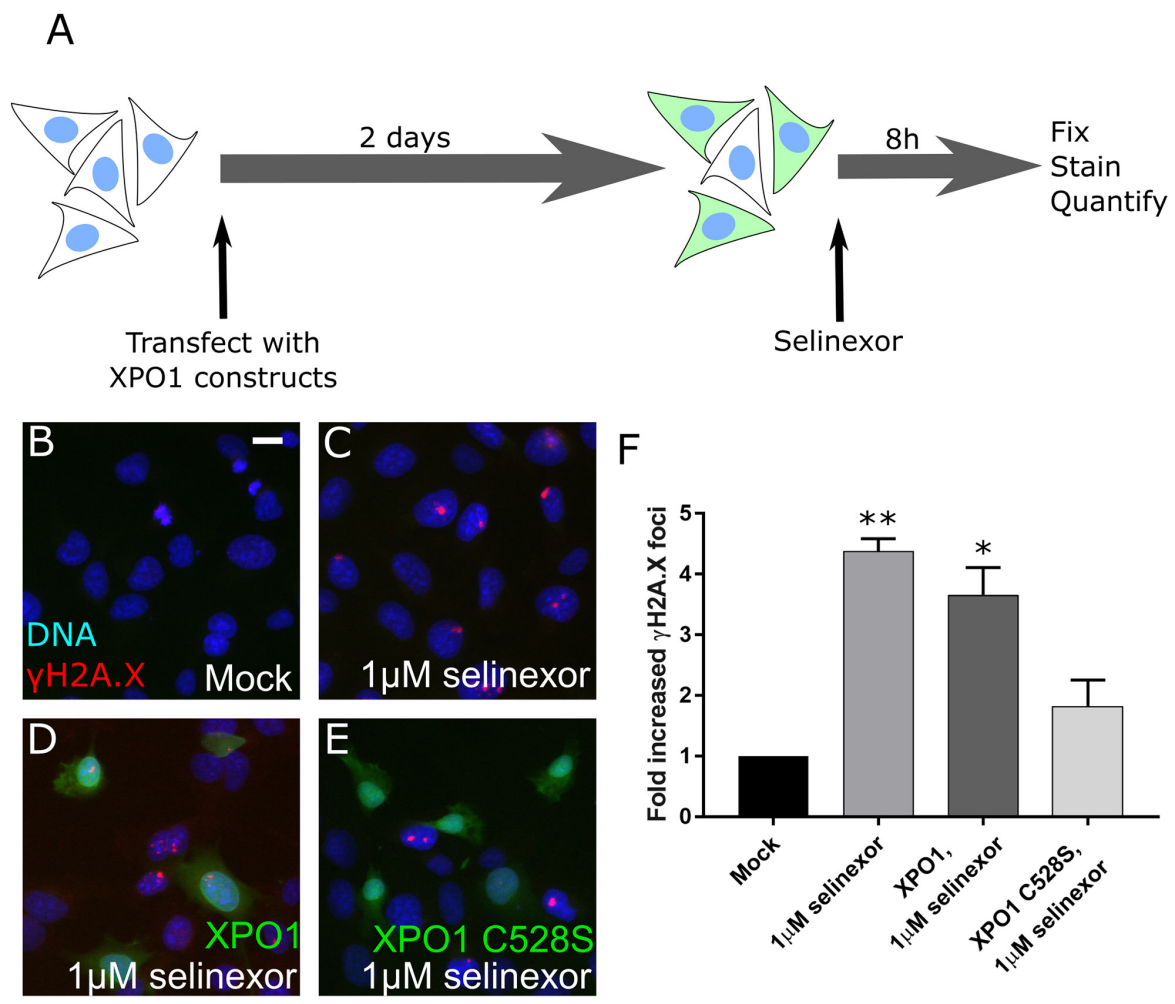
DNA damage foci formation after SINE treatment requires XPO1 binding **(A)** Experimental scheme. Cells are transfected, treated, and the DNA damage formation is quantified. **(B, C)** HT-1080 cells were mock transfected or **(D)** transfected with XPO1-RFP or **(E)** XPO1 C528S-RFP expression plasmids. Cells were treated with DMSO (mock) or 1μM selinexor for 8 hours. Cells were fixed and stained for γH2A.X (red) and DNA (blue). Transfected cells are shown in green. **(F)** The mean fold increase in DNA damage foci over mock was quantified. Error bars are the SEM from two replicate experiments, at least 50 cells scored in each. ** is p<0.01 and * is p<0.05 compared to mock. Scale bar in B = 10μm for all panels.

SINE molecules bind to XPO1 via the cysteine-528 residue [7–9]. To validate that DNA damage formation is specific to XPO1 inhibition by SINE, we transfected cells and expressed XPO1 mutated from a cysteine to a serine at residue 528 (XPO1 C528S). XPO1 C528S cannot bind SINE but is functional to export cargos [21, 22]. Mutant transfected cells were treated for 8 hours with selinexor and the number of cells that form the γH2A.X foci compared to mock transfected cells, transfected cells expressing soluble mRFP, and transfected cells expressing wildtype XPO1 was quantified (Figure 3A). Treated control (1μM selinexor) or XPO1 wildtype expressing (XPO1, 1μM selinexor) cells show a 4-fold increase in γH2A.X foci formation over untreated (mock) cells after SINE treatment (Figure 3B–3D, 3F). Cells expressing the XPO1 C528S mutant show only a 1.5-fold increase in cells with γH2A.X foci (Figure 3E, 3F). XPO1 C528S expression also significantly inhibited γH2A.X foci formation in U2OS cells (Supplementary Figure 2), further demonstrating that DNA damage formation occurs downstream of SINE binding to cysteine-528 of XPO1.

We next characterized and validated the γH2A.X foci in HT-1080 as sites of double-stranded DNA damage. Co-immunofluorescent staining shows the γH2A.X foci also label for 53BP1, NBS1, phospho-(S1981)-ATM and RPA70, which are proteins that mediate the double-stranded DNA damage response (Supplementary Figure 3). Line-scans through representative co-stained foci and plotting of the fluorescent intensity profiles indicates these damage response proteins are strongly localized, supporting these are damaged sites that cells may attempt to repair (Supplementary Figure 3). Standard, low-magnification epifluorescence microscopy (e.g. 20X 0.70 NA air objective), shows the γH2A.X stain as dense, with the structures measuring 1-3 microns in their largest x-y dimension after 8 hours treatment (e.g. Figure 1B or Supplementary Figure 3A). It is unclear whether the structures are a single focus with a large accumulation of γH2A.X staining, or multiple distinct foci that are tightly clustered. High magnification (100X, 1.40NA oil) resolves some structural detail within the γH2A.X foci (Supplementary Figure 4A). Three-dimensional, high-resolution SIM with a lateral resolution of approximately 100nm and axial resolution of approximately 300nm suggests the γH2A.X foci are collections of multiple smaller, distinct, and clustered foci (Supplementary Figure 4B). Next, DNA double-stranded breaks were assessed directly using the neutral comet assay. After 8 hours of treatment with selinexor there is a significant increase in the length of associated comet tails and in the comet tail moment compared to spontaneous damage in control cells, indicating increased double-stranded damage to a similar extent using these measures as is caused by 8 hours of 10μM etoposide (Supplementary Figure 4C-4G).

### DNA damage associates with G1-phase and S-phase cells, and decreased DNA replication

DNA damage can occur throughout the cell cycle, leading to different cellular responses, including cell cycle arrest and death.We first sought to define if γH2A.X foci are cell cycle associated after treatment with selinexor. Using the FUCCI reporter system [10, 23, 24], etoposide (topoisomerase IIα; S/G2-phase inhibition) and PD-0332991 (Cdk4/6; G1-phase inhibition) controls confirm the reporters accurately report on cell cycle phase (Supplementary Figure 5A and [10]). FUCCI expressing HT-1080 cells treated with selinexor for 2, 4, 8, 16, and 24 hours were fixed and stained for DNA and γH2A.X. Nuclei with the γH2A.X foci are classified as red only (G1-phase), red and green (yellow, G1/S-phase), and green only (S/G2-phase) (see Methods); cells in mitosis are excluded from this analysis. At 2 hours treatment, 15-20% of cells show the γH2A.X foci (Figure 2B); at this time, approximately 60% of cells are in G1- or G1/S-phase, regardless of damage status (Supplementary Figure 5A). Over time, the combined percentage of G1- or G1/S-phase cells with foci is relatively constant (Supplementary Figure 5B). In contrast to cells with the γH2A.X foci, cells without DNA damage foci in the same population, shifts persistently and strongly to a G1-phase (red) state over time (Supplementary Figure 5C), in agreement with the cell cycle arrest observed for this cell line previously [10]. We also calculated the fraction of each FUCCI class with γH2A.X foci over time. Between 2–8 hours after treatment, the fraction of G1- and G1/S-phase cells with foci increases from approximately 0.25 to 0.55, before decreasing at 16 and 24 hours (Supplementary Figure 5D). The fraction of S/G2-phase cells with the γH2A.X foci accumulates after 4 hours, and remains elevated at 24 hours when approximately 70% of the total population is in a G1-phase state (Supplementary Figure 5D and 5A). These data support that foci can form in G1- and S-phase, and may associate with prolonged S-phase and/or S/G2-phase arrest. Normal RPE1 cells do not accumulate DNA damage foci (Supplementary Figure 1L) and respond rapidly to selinexor treatment by arresting in G1-phase (Supplementary Figure 6 and Supplementary Video 7), similar to the population in HT-1080 FUCCI that do not form damage (Supplementary Figure 5C).

**Figure 4:**
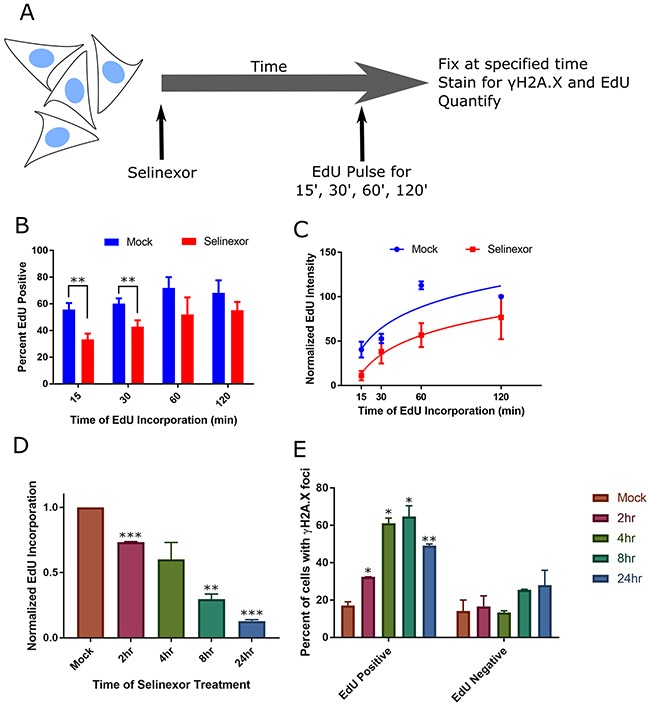
Cells with DNA damage foci associate strongly with S-phase and S-phase progression defects **(A)** Experimental scheme. Cells are pulse-labeled with EdU for varying times at the end of treatment, and quantified. **(B, C)** HT-1080 cells were treated with DMSO (mock) or 1μM selinexor for 8hours (h) and pulse-labeled with EdU for the last 15, 30, 60, or 120 minutes (m). Cells were fixed and costained for γH2A.X, DNA and EdU. Please see Supplementary Figure 7 for representative images. **(B)** The mean percentage of EdU positive cells after 8h selinexor is decreased compared to mock, regardless of EdU pulse length. Error bars are the SEM from three replicate experiments, at least 100 cells scored for each time point. **(C)** The mean integrated EdU signal intensity per cell is decreased after 8h of 1μM selinexor even after long EdU incorporation times. Error bars are SEM from three replicate experiments, at least 100 cells scored for each time point. **(D, E)** HT-1080 cells were treated with 1μM selinexor for 2, 4, 8, and 24h are labeled with EdU for the final 15m of each time point. **(D)** The mean fluorescence of EdU decreases as the duration of treatment increases. Error bars are the SEM from two replicate experiments, at least 100 cells measured for each time point. **(E)** The population of analyzed cells was divided into two groups, EdU positive and EdU negative. γH2A.X foci were identified and the percentage of cells in each group with foci was quantified. EdU positive cells show damage foci more frequently than EdU negative cells. After 8h of selinexor treatment, 70% of EdU positive cells show foci compared to 25% of EdU negative cells. Error bars are the SEM from two replicate experiments, at least 100 cells measured for each time point. *** is p<0.001, ** is p<0.01 and * is p<0.05.

DNA damage can cause –or– be caused by S-phase progression defects [17]. We evaluated a potential relationship between DNA damage foci and S-phase after treatment with selinexor (Figure 4A). Following 8 hours of selinexor treatment, fewer HT-1080 cells are actively replicating their DNA and replication is significantly decreased based on quantification of the incorporation of the nucleotide 5-ethynyl-2′ deoxyuridine (EdU) - even after 2 hours of EdU incubation (Figure 4B, 4C, Supplementary Figure 7). Cells treated in a time course with selinexor, followed by a 15min EdU pulse, were co-stained for γH2A.X. The mean incorporation of EdU per cell begins to decrease after 2 hours of selinexor exposure (Figure 4D), suggesting a rapid impact on S-phase progression, and by 24 hours, it is negligible. EdU – γH2A.X foci correlation analysis indicates a positive association between positive, but decreased EdU labeling and the presence of γH2A.X foci, that increases until 8 hours after treatment (Figure 4E). EdU negative cells in the same treated population show no strong association with γH2A.X foci status, but it does appear to somewhat increase over time compared to the EdU negative cells in the mock treated population (Figure 4E), indicating dead cells or that at least some cells with foci become arrested. In U2OS cells, selinexor treatment also results in decreased DNA replication based on EdU incorporation, and γH2A.X foci associate strongly with S-phase status after 8 hours of treatment (Supplementary Figure 8); foci are present in approximately 30% of cells fixed after 8 hours of treatment. EdU incorporation in cells is nearly absent altogether after a 24 hour treatment (Supplementary Figure 8A). These results in U2OS are consistent with HT-1080. Taken together, the FUCCI and EdU data indicate an association between DNA damage formation and the cell cycle, but the precise timing of the damage cannot be resolved nor does it allow for the direct determination of cell fate in cells with and without damage.

### Longitudinal tracking shows that nearly 50% of all cells become damaged, mostly in G1- and S-phase, and >90% of cells damaged in G1-phase subsequently die

**Figure 5:**
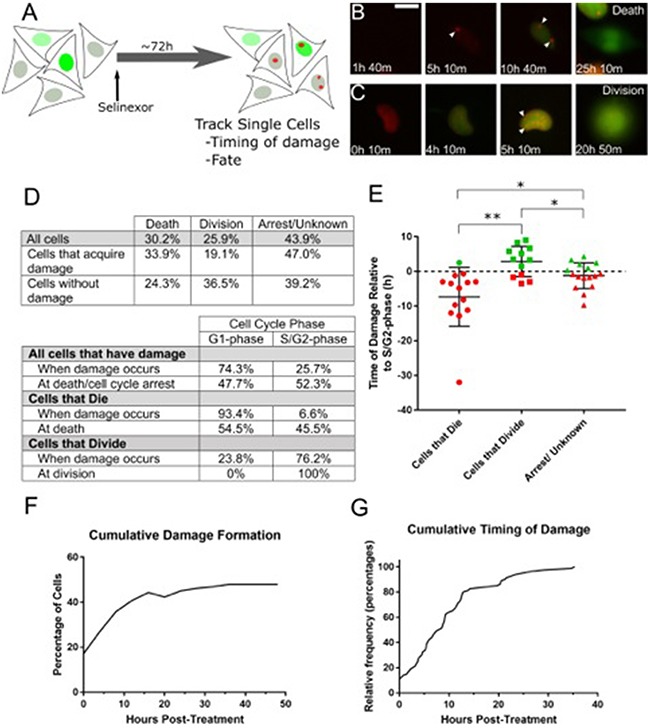
Live cell tracking of SINE treated cells reveals cell cycle associated DNA damage and cell fates **(A)** Experimental scheme. HT-1080 cells that express mAG-hGem(1/110) and mCherry-BP1-2 were treated with 1μM selinexor and imaged every 10 minutes (m) for 72 hours (h). DNA damage foci formation, cell cycle stage and cell fate were analyzed. (B, C) Representative examples of cells that acquire DNA damage foci are presented. **(B)** This cell acquires damage 5h 10m after treatment. The cell then enters S-phase (accumulation of mAG-hGem(1/110) probe) at 10h 40m and dies at 25h 10m. **(C)** The second example enters S-phase at 4h 10m, acquires damage foci at 5h 10m and progresses to cell division at 20h 50m. White arrows indicate DNA damage foci. **(D)** Tables summarize the fate and cell cycle stages of 189 individually tracked cells. Over 70% of cells that become damaged, are damaged in G1-phase (absence of mAG-hGem(1/110) probe). For cells that die, over 90% are damaged in G1-phase, those cells that acquire damage and progress to cell division become damaged in S/G2-phase. **(E)** Cells that progress to S/G2-phase were analyzed to determine the timing of DNA damage accumulation in relation to the S/G2-phase transition and cell fate. The dotted line is S/G2-phase entry, designated time=0. Each point is an individual cell. The bars are the mean with standard deviation. Cells that become damaged and die in S/G2-phase are typically damaged several hours before S/G2-phase entry, where those that become damaged but divide are damaged shortly before or after S/G2-phase entry. ** is p<0.01 and * is p<0.05. **(F)** Cumulative damage formation within the entire tracked population. Nearly 50% of all cells acquire damage, mostly by 15h after treatment, and approximately 36% after 8h, in good agreement with fixed cell experiments. **(G)** The cumulative timing of damage for all cells that acquire damage is displayed. Damage formation is rapid until approximately 15h and slower thereafter. Scale bar in B = 10μm for all panels.

Fixed cell experiments show DNA damage increases within hours after treatment with 1μM SINE. For HT-1080, the peak percentage of cells with damage occurs at 8 hours and remains elevated at 24 hours, and the damage associates with G1- and S-phase (Figure 2, 4 and Supplementary Figure 7). To precisely define the timing of DNA damage formation with regard to G1- and S-phase directly in the same cell, we employed an HT-1080 reporter cell line stably co-expressing the double-stranded DNA damage probe, mCherry-BP1-2 (red, see Material and Methods for detail), and the S/G2-phase FUCCI probe, mAG-hGem(1/110) (green), and time-lapse microscopy and longitudinal tracking was performed (Figure 5A) [10, 25]. After treatment with SINE, nearly 75% of cells that form DNA damage do so in G1-phase (absence of green signal); the remaining 25% are in S/G2-phase (green) (Figure 5B versus 5C, and Figure 5D). Cells that acquire DNA damage in G1/S-phase (yellow in FUCCI system, Supplementary Figure 5) are represented in the S/G2-phase population using this reporter cell line. Longitudinal tracking reveals that close to 50% of all treated cells acquire damage within the first 24 hours after treatment with SINE, and approximately half of this occurs by 8 hours (Figure 5F). Timing analysis of damage formation in the damaged population indicates a rapid increase to approximately 15 hours after treatment, in support of a tight association with the cell cycle (Figure 5G). The cell cycle association of DNA damage and fraction of treated cells with damage foci over time suggest these cells are gradually lost and/or that the damage is repaired (Figures 2, 5 and Supplementary Figure 5 and 8).

When SINE treated cells acquire DNA damage they are not dead. Rather, cells with DNA damage may undergo death, cell cycle arrest or senescence, or they may continue proliferating. Longitudinal tracking of treated HT-1080 mAG-hGem(1/110)/mCherry-BP1-2 cells shows that by 48 hours after treatment, 30% of all cells died and 26% divided (Figure 5D); over 40% of cells are classified as arrest/undetermined due to the end of imaging or movement from the analysis field; previous work indicates these cells may remain arrested [10]. The treated cell population can be parsed into populations that become damaged versus those that do not. Cells that acquire damage are more likely to die (33.9%) than to progress to cell division (19.1%) (Figure 5D). By comparison, treated cells that do not acquire damage divide more frequently (36.5%) than they die (24.3%). SINE treatment causes death with and without damage, but death is elevated in cells that become damaged (33.9% versus 24.3%).

Of the damaged cells that die, greater than 90% show damage initially in G1-phase (Figure 5D); 58.4% remain in G1-phase and die and 41.6% progress to S/G2-phase and die (e.g. Supplementary Videos 3 and 2, respectively). Of the damaged cells that progress to cell division, 76.2% were damaged in S/G2-phase (Figure 5C, 5D, Supplementary Video 5). Next, we characterized the timing of DNA damage with relation to S-phase entry and cell fate for cells that form damage and progress to S/G2-phase (Figure 5E). For cells that form damage and die in S/G2-phase, damage most often occurs in G1-phase, 7-8 hours on average before entering S-phase (increasing mAG-hGem(1/110) probe). For cells that form damage and progress to cell division, DNA damage on average forms 2-3 hours after S-phase entry. The timing of DNA damage is on average 1-2 hours before entering S-phase for cells that arrest or whose fate could not be determined (Figure 5E). The ultimate fate of cells that acquire DNA damage and maximizing their death is an important consideration for how to most effectively use SINE against cancer.

### Multiple classes of agents that compound DNA damage show combination effects with selinexor

**Figure 6:**
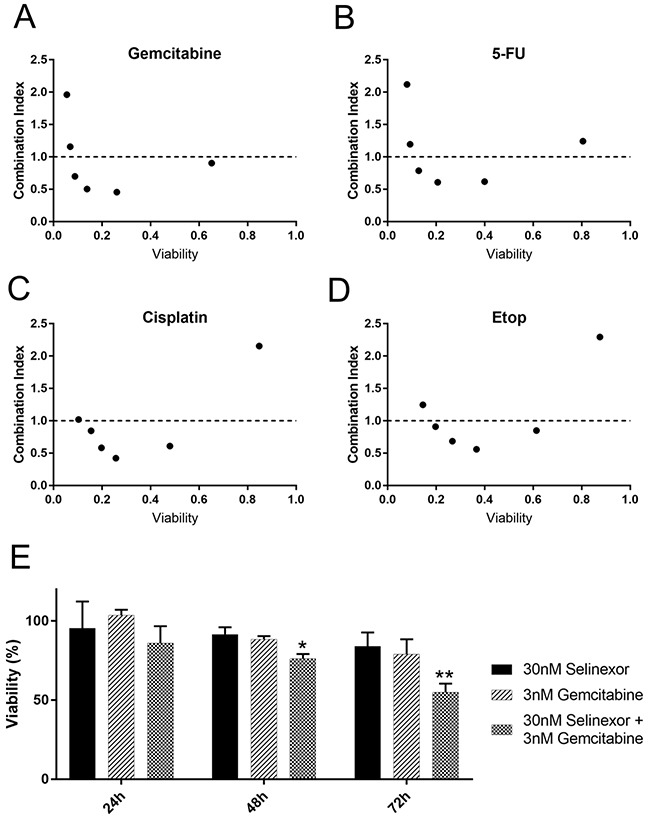
Selinexor combines synergistically with different classes of DNA damage agents HT-1080 cells were treated with selinexor and the FDA approved therapeutics in equimolar concentrations **(A-D)** Relative cell survival was detected with CellTiter-Glo after 72 hours (h). Combination indices for each combination were calculated using the median effect model. The dotted line represents a combination index of 1. Points less than 1 are synergistic whereas points greater than 1 are antagonistic. **(E)** A time course with selinexor or gemcitabine alone and in combination was performed at half the EC_50_ concentration. Survival at each time point is determined via normalization to DMSO (mock) treated wells. A significant decrease in survival compared to mock treated is seen at 48 and 72h post treatment in the combination. Error bars are SEM from 3 experiments. ** is p<0.01 and * is p<0.05.

DNA damage is a critical avenue to clinical efficacy for many cancer treatments, especially in combination chemotherapies that compound DNA damage to enhance the anti-cancer effect [26–28]. Nearly 50% of all cells form DNA damage after treatment with SINE (Figure 5F). While cells that acquire damage show modestly elevated frequency of death than their undamaged counterparts, over 60% arrest or progress to cell division (Figure 5D). We tested equimolar combinations of selinexor with multiple different DNA damage agonists in an attempt to find combination effects that significantly decrease cell survival. We used nucleoside analogs, a platinum-based DNA intercalator, a topoisomerase IIα poison, and a poly-ADP-ribose polymerase-1 (PARP1) inhibitor that are all approved for the treatment of human cancers in combination with other agents (Figure 6 and Supplementary Figure 9 and 10). Further, clinical trials are planned that combine selinexor with these or highly related chemotherapy compounds (ClinicalTrials.gov).

The median effect model was used to calculate the combination effects. Both CompuSyn and an R based analysis package (see methods) were used to calculate the combination effect indices using ATP as a surrogate for cell survival (Figure 6 and Supplementary Figure 10). Results from both methods were the same, and results using CompuSyn are shown as it is widely available. Applying the guidelines of Chou and Martin [29] regarding drug combination effects, there is significant synergy (combination index <1.0) of selinexor with each of these agents in at least some equimolar combinations (Figure 6A–6D and Supplementary Figure 10B). The concentrations of selinexor used are 31.25nM-1μM in each combination series. The other compounds were used at equimolar ratios depending on the effect of the compound alone (1:10, 1:1 or 10:1, see Supplementary Figure 9 and 10).

The nucleoside analogs gemcitabine and 5-FU show strikingly similar combination effects across the dose combinations tested (Figure 6A, 6B). Gemcitabine shows combination indices <1.0 for the four lowest concentration combinations. We chose concentrations that were approximately half the EC50 to characterize the selinexor– gemcitabine combination over time (30nM selinexor, 3nM gemcitabine) (Figure 6E, Supplementary Figure 9A). As single agents, there is no significant decrease in cell survival by 72 hours compared to untreated. The combination has a significant decrease in viability compared to untreated cells at, with an approximate 25% decrease in survival at 48 hours, and nearly a 50% decrease at 72 hours (Figure 6E).

The DNA intercalator cisplatin combines well with selinexor at four of the tested combinations (Figure 6C). The topoisomerase IIα poison, etoposide has been combined with selinexor in chronic lymphocytic leukemia [11] and acute myeloid leukemia [27], and also shows combinations effects here with HT-1080 cells (Figure 6D). The EC50 for cell survival for cisplatin and etoposide are approximately 498.7nM and 59.7nM, respectively (Supplementary Figure 5). The PARP1 inhibitor olaparib combines well with DNA damaging treatments and other chemotherapies [30, 31] and shows combination effects with selinexor in triple negative breast cancers cells independent of BRCA1 status [32]. When combined with selinexor in HT-1080 cells (BRCA1 wildtype, [33]), olaparib shows combination effects in five different combinations; olaparib also combines well with x-irradiation as a positive control, although combination indices cannot be calculated in this case (Supplementary Figure 10A-10C). Taken together, selinexor combines well with chemotherapy agents that each induce double-stranded DNA damage through distinct molecular mechanisms (gemcitabine, 5-FU, cisplatin, and etoposide) and that perturb DNA repair and apoptosis signaling (olaparib).

## DISCUSSION

Treatment of cells with SINE compounds results in multiple cell fates, including cell cycle arrest, cell cycle progression defects, and apoptosis [7, 8, 10]. The mechanisms by which SINE compounds exert these effects need to be understood if we are to best utilize these agents to treat cancers. The sequestration of some XPO1 cargos, the functions of some proteins, and gross cell fates after treatment with SINE indicate that nuclear export is blocked rapidly and the cell responses are highly complex [6, 10, 20, 34]. For example, recent studies document decreased ribosome biogenesis [35], disrupted nuclear architecture of telomeres [36], synthetic lethality with oncogenic K-Ras [20], and NFκB/IκB regulation after treatment with SINE [34].

Here, we show that double-stranded DNA damage occurs in some cells within hours of treatment with SINE (Figures 1, 2) and longitudinal tracking experiments in one cell model indicate nearly half of the population becomes damaged by 24 hours and correlates strongly with eventual cell death and arrest, particularly if the damage occurs when cells are in G1-phase (Figure 5). Over time, cells with DNA damage show a strong association with S-phase based on positive, but decreased EdU staining (Figure 4 and Supplementary Figures 7, 8). The DNA damage foci stain for multiple DNA damage repair proteins consistent with double-stranded damage (Supplementary Figure 3). Indeed, based on single cell tracking (Figure 5D), while 34% of cells with damage progress to cell death, 66% appear to either repair the foci in a protracted S/G2-phase (and divide) (e.g. Supplementary Video 5) or remain in an arrested state (Supplementary Video 6).

High-magnification, high-resolution microscopy and the neutral DNA comet assay reveal there are multiple, clustered DNA damage foci after treatment with SINE (Supplementary Figure 4); a small number of breaks does not generate tails in the comet assay. Notably, the population of HT-1080 cells at 8 hours of treatment with SINE that is used in the comet assay contains very few apoptotic cells [10], indicating the tails are not due to DNA fragmentation associated with cell death. Cells expressing the functional XPO1 C528S point mutant that cannot bind SINE show decreased DNA damage foci formation compared to cells expressing wildtype XPO1 (Figure 3 and Supplementary Figure 2); DNA damage formation is not completely inhibited by expression of XPO1 C528S, likely due to the continued expression of normal XPO1. These data indicate that SINE binding to XPO1 is causal to a subsequent mechanism of DNA damage and that the SINE molecules themselves are not directly causing the damage.

DNA damage in general can result from many different mechanisms. The data indicate that multiple, clustered, double-stranded breaks occur within hours after treatment with SINE (Figure 2 and Supplementary Figure 4). Given the pattern of the foci and timing of their formation it is unlikely that global DNA replication defects are responsible. It is possible that an early replicating gene cluster at the G1/S-phase transition is prone to damage after XPO1 inhibition, but attempts to co-localize the damage foci with EdU shows little if any colocalization (not shown). DNA damage foci localized at telomeres and centromeres are known [37, 38]. Damage localized with centromeres is reported to be associated with mitotic defects [38] and XPO1 does have reported roles at centrosomes and in chromosome attachment to the centromere that could potentially perturb mitosis when inhibited [39, 40]. However, the clustered foci studied here after treatment with 1μM selinexor are observed to form predominantly in G1-phase cells without any obvious association with mitosis (Figure 2, 5, Supplementary Video 4). Telomere dysfunction-induced foci (TIFs) [37] are found in small numbers in some cell lines growing in culture, label with double-stranded break markers [41] and associate with decreased cell proliferation and increased senescence [42]. It is conceivable that SINE treatment impacts telomere signaling or length, given that a component of the telomere cap, TPP1, may bind XPO1 [43]. When TPP1 dominant inhibitors are expressed, numerous TIF form that are scattered throughout the nucleus [43]. Further, HT-1080 express telomerase and telomere length is stable [44], and uncapping by telomestatin did not cause growth defects until >4 days [45]. The acute nature of the experiments here and the pattern of damage staining indicate a telomere-based mechanism is unlikely. Future work will focus on the molecular mechanism of DNA damage formation.

DNA damage foci are observed in multiple cancerous cell lines after selinexor treatment (Supplementary Figure 1). Interestingly, two non-cancer cell lines, RPE1 and MSCs, show no appreciable increase in damage foci (Supplementary Figure 1). The identification of underlying sensitizing factors to DNA damage formation after treatment with selinexor will make an important future contribution to how these molecules work on cells. Based on experiments with RPE1 and MSC cells, one possibility is that at least some normal cells respond very rapidly to SINE treatment and arrest in early G1-phase (e.g. RPE1 FUCCI, Supplementary Figure 6 and Supplementary Video 7), prior to DNA damage formation, and show little death. This idea agrees with earlier observations showing less cytotoxicity of normal cells to selinexor [8, 12, 46]. Taken together, there is likely a common mechanism underlying the DNA damage, but it does not mean that the foci form at the same frequency or with the same kinetics in all cell lines. This may be especially true given the G1- and G1/S-phase association of the damage, that cell cycle progression is different between cell lines, and that some cells are more capable of strong G1-phase arrest and DNA repair than others based on signaling pathways. It is possible that overall response to selinexor response is directly affected by the functionality of various XPO1 cargos, such as p53 [47, 48]. Due to p53 being an XPO1 cargo involved in cell cycle arrest and cell death after DNA damage, it will be important to investigate p53 loss of function as it relates to the DNA damage and cell fate observed here. Other mechanisms in cancer cells may also impact response and fate after selienxor treatment. For example, oncogenic Ras signaling, which impacts telomere dysfunction, ribosome biogenesis, and DNA replication, has been observed to decrease cell survival after selinexor treatment [20, 49, 50].

**Figure 7:**
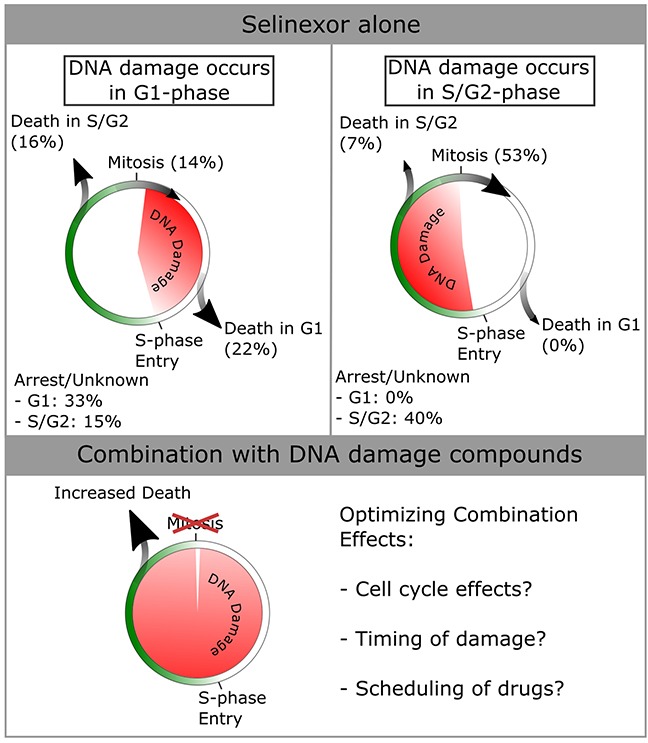
Summary of cell cycle -associated cell fates after DNA damage in HT-1080 Data from Figure 5 were used to compile cell fates dependent on when damage occurs in the cell-cycle. More death is seen in cells that acquire damage in G1-phase (total of 38% versus 7% for damage in S/G2-phase). Cells that accumulate damage in S/G2-phase most often progress through mitosis (53% versus 14% for damage in G1-phase). Given the synergistic effects of combining selinexor with DNA damage agonists [see Figure 6], cell death is increased across the entire population. The mechanisms of optimizing combinatorial effects should be studied, and are hypothesized to be cell cycle effects caused by either/both compounds, the timing of DNA damage within the cell cycle, and/or the scheduling of drugs to maximize the combinatorial effects.

DNA damage after treatment with SINE correlates with cell death in 34% of the cells, other cells appear to repair the damage and continue proliferation or undergo cell cycle arrest, and still others show no DNA damage, but also die or arrest (Figure 5, e.g. Supplementary Videos 3, 4, and 6). Cell fate appears to be dependent on the timing of DNA damage, with death occurring most often in cells that acquire damage in G1-phase (38%) compared to those that acquire damage in S/G2-phase (7%) (Figure 7). These features of the response to SINE may enable the combination effects found when SINE is combined with different classes of DNA damage agents. Combination treatments are an essential part of anti-cancer therapies and are an important part of future research (Figure 7). Of note, gemcitabine, platinum-based agents, topoisomerase inhibitors and PARP1 inhibitors are each being evaluated with selinexor in clinical trials (NCT02178436, NCT02269293, NCT02299518, NCT02419495, ClinicalTrials.gov). Our data suggest that SINE combined with DNA damaging drugs may significantly decrease cell survival and, optimistically, some combination will result in a stronger initial response in cancer therapy.

## MATERIALS AND METHODS

### Cell lines and plasmids

HT-1080 (ATCC) are grown in MEM with Earle's salts (Corning; 10-010-CV), sodium pyruvate (Sigma), non-essential amino acids (Sigma), penicillin/streptomycin (Sigma; P/S), and 10% FBS (Sigma). U2OS are grown in McCoys5a (Corning; 10-050-CV) with 10% FBS and 1% P/S. MCF7 are grown in RPMI (Corning; 10-040-CV), 10% FBS, and 1% P/S. HeLa, PANC-1 and human mesenchymal stem cells (MSC) are grown in DMEM (Sigma; D6429-500ML), 10% FBS, and 1% P/S. RPE1 are grown in DME/F-12 1:1 (Hyclone; SH30023.01), 10% FBS, and 1% P/S.

MSCs are obtained by a procedure adapted from Ahfeldt et al. [51]. hiPSCs were cultured feeder free on Matrigel (Corning; 356234) in chemically defined E8 medium (Thermo; A1517001). For differentiation of hiPSCs into embryoid bodies, hiPSCs were disaggregated with 0.5mM EDTA into small clumps containing 5–10 cells and transferred to low-adhesion plastic 6-well dishes (Costar Ultra Low Attachment; Corning Life Sciences) in growth medium containing DMEM, 15% KOSR (Thermo; 10828010) and 1% GlutaMAX (Thermo; 35050061). After 7 days, embryoid bodies were collected and replated on gelatin-coated 6-well dishes in DMEM, 10% FBS, 1% GlutaMAX, 1% P/S. Upon confluency, cells were trypsinized in 0.25% trypsin:EDTA (Sigma; T4049) and replated on cell culture dishes and maintained as described above.

The HT-1080 mAG-hGem(1/110)/mCherry-BP1-2 expressing cell line was engineered by transfecting an HT-1080 mAG-hGem(1/110) cell line with the mCherry-BP1-2 expression plasmid (FuGENE 6, Promega; E2691) and selection in 1μg/ml puromycin. Cell lines expressing both probes were obtained via clonal selection in 96 well plates. The mAG-hGem(1/110) plasmid is from Sakaue-Sawano *et al*. [23] via material transfer agreement. The mCherry-BP1-2 plasmid expresses a peptide fragment of the DNA damage response/transcriptional regulator protein 53BP1 that includes the γH2A.X binding domain and a mutated, non-functional Tudor domain, but lacks both BRCT domains. The peptide retains the capacity to localize to sites of double-stranded DNA damage [41]. mCherry-BP1-2 pLPC-Puro was a gift from Titia de Lange (Addgene plasmid # 19835). Detection of DNA damage response using mCherry-BP1-2 was confirmed by treating expressing cells with 10μM etoposide and time-lapse microscopy (Supplementary Video 1). The XPO1- and XPO1 C528-RFP expression plasmids are a gift from Yossi Landesman.

### Antibodies, immunofluorescence, and stains

Phospho-serine-139 H2A.X (γH2A.X) mouse monoclonal (Millipore JBW101) and rabbit monoclonal (Cell Signaling 20E3) are used at 1:500 dilution. Other antibodies are: 53BP1 (Cell Signaling 4937, 1:200), NBS1 (Novus Biologicals 100-143. 1:500), pATM Ser1981 (Millipore 05-740. 1:1000), and RPA-70 (Santa Cruz 28304. 1:200). Goat and donkey anti-mouse or anti-rabbit secondary antibodies conjugated to AlexaFluor 488, 568, or 647 are from Invitrogen and used at 1:500 dilution. Cells were grown on #1.5 glass coverslips (VWR 48366-227). Cells were fixed in 3.7% formaldehyde in PBS (pH 7.4) for 20 minutes at room temperature, washed at least 3 times in PBS, permeablized in 0.5% Triton X100 (Sigma) in PBS, washed at least 3 times in PBS, blocked in 4% BSA in PBS for 60 minutes at room temperature, incubated in primary antibody diluted in 4% BSA/PBS for 60 minutes at room temperature, washed at least 3 times in PBS, incubated in secondary antibody diluted in 4% BSA/TBS for 60 minutes at room temperature, washed at least 3 times in PBS, and counterstained with 1μM DAPI for 5 minutes at room temperature, washed in distilled water, and mounted in ProLong Gold or Prolong Diamond antifade reagent (Invitrogen) on glass microscope slides (VWR, 16004-422).

### Small molecules and treatment of cells

The SINE compounds KPT-185, KPT-330 (selinexor), and KPT-8602 and the inactive KPT-185 trans isomer, KPT-301, are from Karyopharm Therapeutics, Inc. (Newton, MA), and are prepared in anhydrous DMSO (Sigma, Hybrimax) and used at the concentrations indicated. Etoposide and gemcitabine are from Selleckchem, dissolved in DMSO, and used at the concentrations indicated. Cisplatin (Sigma) is dissolved in dimethylformamide (Sigma), and used at the concentrations indicated. 5-Fluorourcil (5-FU) (Sigma) is dissolved in DMSO and used at the concentrations indicated. Olaparib (LC laboratories) is dissolved in DMSO and used at the concentrations indicated. For FUCCI fixed cell experiments, 10μM Cdk4/6 inhibitor PD-0332991 (Selleckchem) for 16 hours is used as a G1-phase arrest standard, and 10μM etoposide (Selleckchem) for 8 hours is used as an S/G2-phase arrest standard as previously described for HT-1080 cells[10]. Cells are approximately 70% confluent at the time of drug treatments.

### Microscopy, FUCCI scoring, cell tracking, and quantification

Fixed cell, immunofluorescence microscopy was performed using an inverted Olympus IX81 microscope with Prior Lumen200 Pro metal halide lamp, Hamamatsu ORCA R2 CCD camera, motorized Prior ProScan III stage, and 20X 0.70NA, 40X 0.75NA, and 100X oil immersion 1.40NA objectives using optical filters for DAPI (Chroma), Alexa488/EGFP (Chroma), Alexa568/mCherry (Chroma) and Alexa647/Cy5 (Semrock). High-resolution, structured illumination microscopy (SIM) was performed using a Nikon A1/N-SIM microscope with 100X oil immersion 1.49NA TIRF objective and 405, 488, 561, and 647 lasers and optical filters from Chroma. SIM images were reconstructed using Nikon Elements software. Two investigators scored the FUCCI status in fixed cell experiments. Briefly, nuclei (DAPI) were identified using the Analyze Particles tool in FIJI (NIH). Fluorescence intensity values in the red and green channels were measured and based on signal over background cells were scored as G1- (red), G1/S (yellow), or S/G2-phase (green). For DNA damage foci in fixed cells, the “Find Maxima” tool in FIJI using appropriate thresholding based on positive control cells treated with 10μM etoposide to isolate DNA damage foci above background was used, and cells were scored as positive or negative. Live-cell microscopy was performed using an inverted Olympus IX81, 20X 0.70NA objective, and stage-top incubation chamber (InVivo Scientific) as described previously (see [10, 21]). Autofocusing was performed using phase-contrast. To minimize acquisition delay between green and red channels, EGFP/mCherry dual optical filters (Semrock) and filter wheels were used. Control conditions are included in each experiment to confirm normal growth. Two investigators independently tracked all live cells. For DNA damage foci formation, puncta matching the properties of those found in fixed cells were identified by eye and validated by a second investigator. Each cell was tracked longitudinally and fates were defined as; 1) death, cell rounding accompanied with blebbing and cell fragmentation, 2) arrest, cells remain in interphase, and 3) cell division, cell enters mitosis and completes division. The cell numbers analyzed in each case are provided in the respective figure legends.

### Neutral DNA comet assay

The Trevigen DNA comet assay kit was used following the manufacturer's protocol. Briefly, drug treated cells are resuspended with 0.5% Trypsin and 3×10^6^ cells/ml were suspended into low melting point agarose. Cells were placed onto glass slides provided with the kit. Prepared slides were placed in 1X TAE running buffer and electrophoresed at 22 volts for 35 minutes, per the manufacturer's protocol. DNA was stained using Sybr Gold (Invitrogen). Glass coverslips were mounted on top of the sample using Prolong Gold anti-fade reagent. DNA comets were imaged using an Olympus IX81 inverted epifluorescence microscope and 10X 0.40NA objective with a YFP filter set (Omega). Tail length and tail moment were calculated using the ImageJ plugin OpenComet [52]. All tail-lengths and tail moments are plotted using box and whisker plots, the median is indicated. Significant differences between the populations are calculated using a two-tailed student's T-test. Comet number is >100 for each condition from two experiments.

### Drug combination effects

Selinexor was combined with gemcitabine, 5-FU, etoposide, cisplatin, or olaparib. Briefly, 500-1000 HT-1080 cells were plated into 96 well white-walled plates (Thermo Scientific) with glass or optical plastic bottoms, grown overnight, and treated with the indicated conditions for 72 hours. ATP luminescence (CellTiter-Glo 2.0, Promega) was read using a Biotek plate reader within 10 minutes of sample preparation. Effects on cell survival over equimolar dose combinations were calculated using the median effect model [53, 54]. Statistical modeling of combination effects was determined using a script in R based on previous methods, and using the software package CompuSyn [29, 55, 56]. For the selinexor and gemcitabine combination, luminescence values were also measured at 24, 48, and 72 hours. For an x-irradiation control, cells in 96 well plates were exposed to 3 Gy.

## SUPPLEMENTARY FIGURES AND VIDEOS
















