# Supplementary material for: Inhibition of exportin-1 function results in rapid cell cycle-associated DNA damage in cancer cells
Source: Oncotarget. 2017 Apr 12;8(24):39460–75. doi: 10.18632/oncotarget.17063 (PMC5503625; doi:10.18632/oncotarget.17063)
Supplement: Supplementary file 1 [file oncotarget-08-39460-s001.pdf]

Inhibition of exportin-1 function results in rapid cell cycle-associated DNA damage

SUPPLEMENTARY MATERIALS

SUPPLEMENTARY FIGURES AND VIDEOS

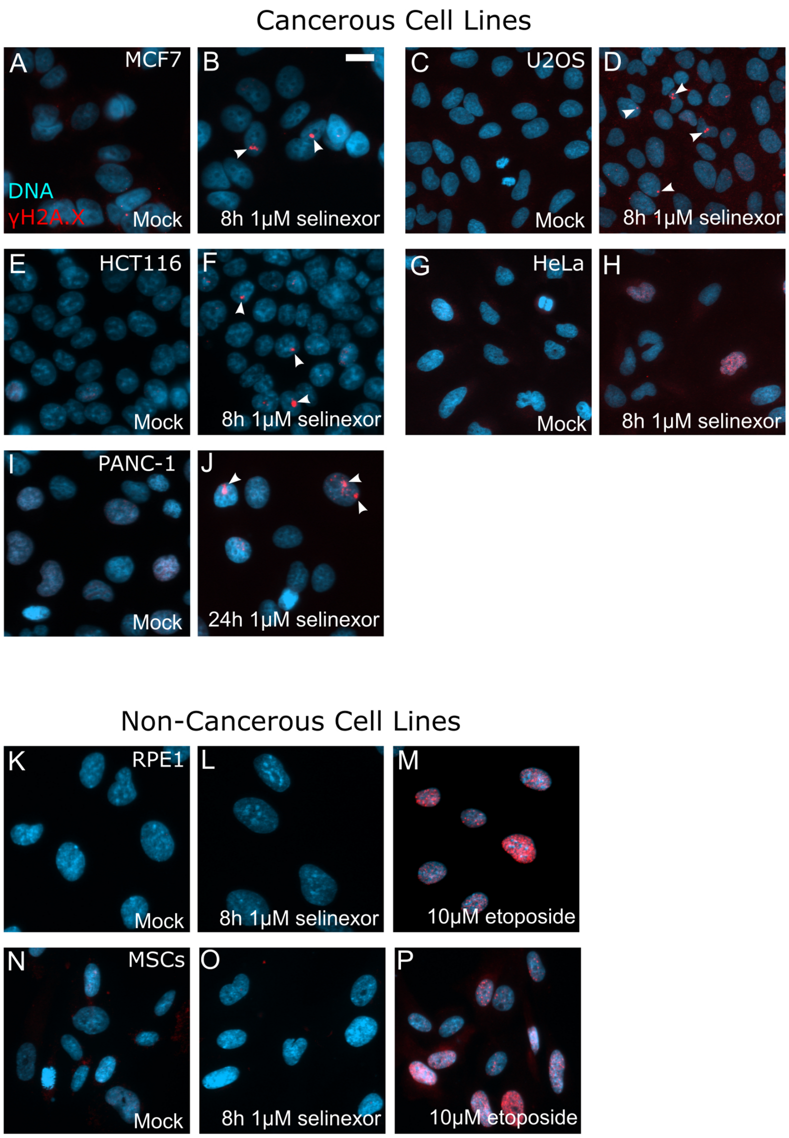

Supplementary Figure 1: Several cancer-derived cell lines show increased, large DNA damage foci after selinexor treatment while non-cancer cells do not. Cells were treated with DMSO (control) or 1μM selinexor for the time indicated and stained for γH2A.X (red) and DNA (blue). White arrows indicate DNA damage foci. **(A, B)** MCF7 breast cancer derived cells show foci after 8 hours (h) treatment. **(C, D)** U2OS bone cancer derived cells show foci after 8h treatment. **(E, F)** HCT116 colon cancer derived cells show foci after 8h treatment. **(G, H)** HeLa cervical carcinoma cell show increased damage after 8h treatment. **(I, J)** PANC-1 pancreatic cancer derived cells show foci after 24h treatment. **(K-M)** RPE1, non-transformed human telomerase immortalized cells derived from retinal epithelium, show no strong foci formation after 8 hours and 24 hours (not shown) treatment. DNA damage is present after 8 hours of 10μM etoposide. **(N-P)** Human mesenchymal stem cells (MSC) show no obvious increase in γH2A.X foci formation after8h and 24h (not shown). DNA damage is present after 8h of 10μM etoposide. Scale bar in B = 10μm for all panels.

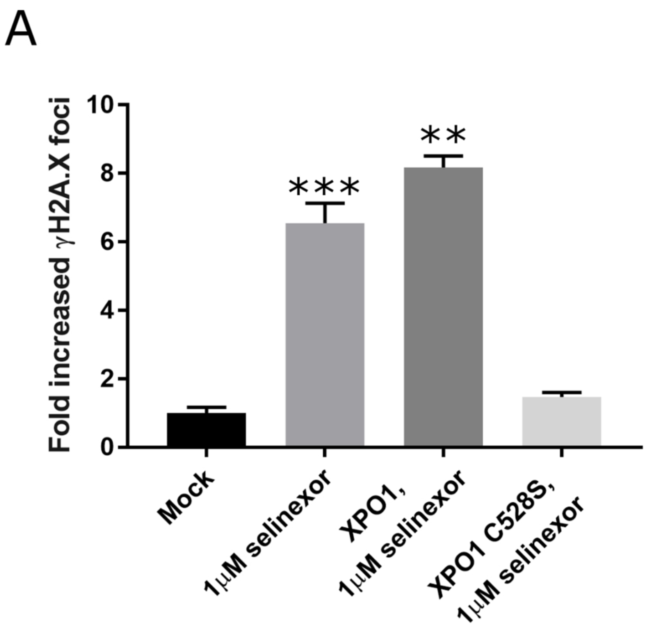

Supplementary Figure 2: SINE treatment resulting in DNA damage foci requires XPO1 binding in U2OS cells. Experimental scheme is as Figure 3A. U2OS cells were mock transfected or transfected with XPO1-RFP or XPO1 C528S-RFP expression plasmids. Cells were treated with DMSO (mock) or 1μM selinexor for 8 hours. Cells were fixed and stained for γH2A.X and DNA. **(A)** The mean fold increase in DNA damage foci over mock was quantified. Error bars are the SEM from two replicate experiments, at least 150 cells scored in each. *** is $p<0.001$ and ** is $p<0.01$ compared to mock.

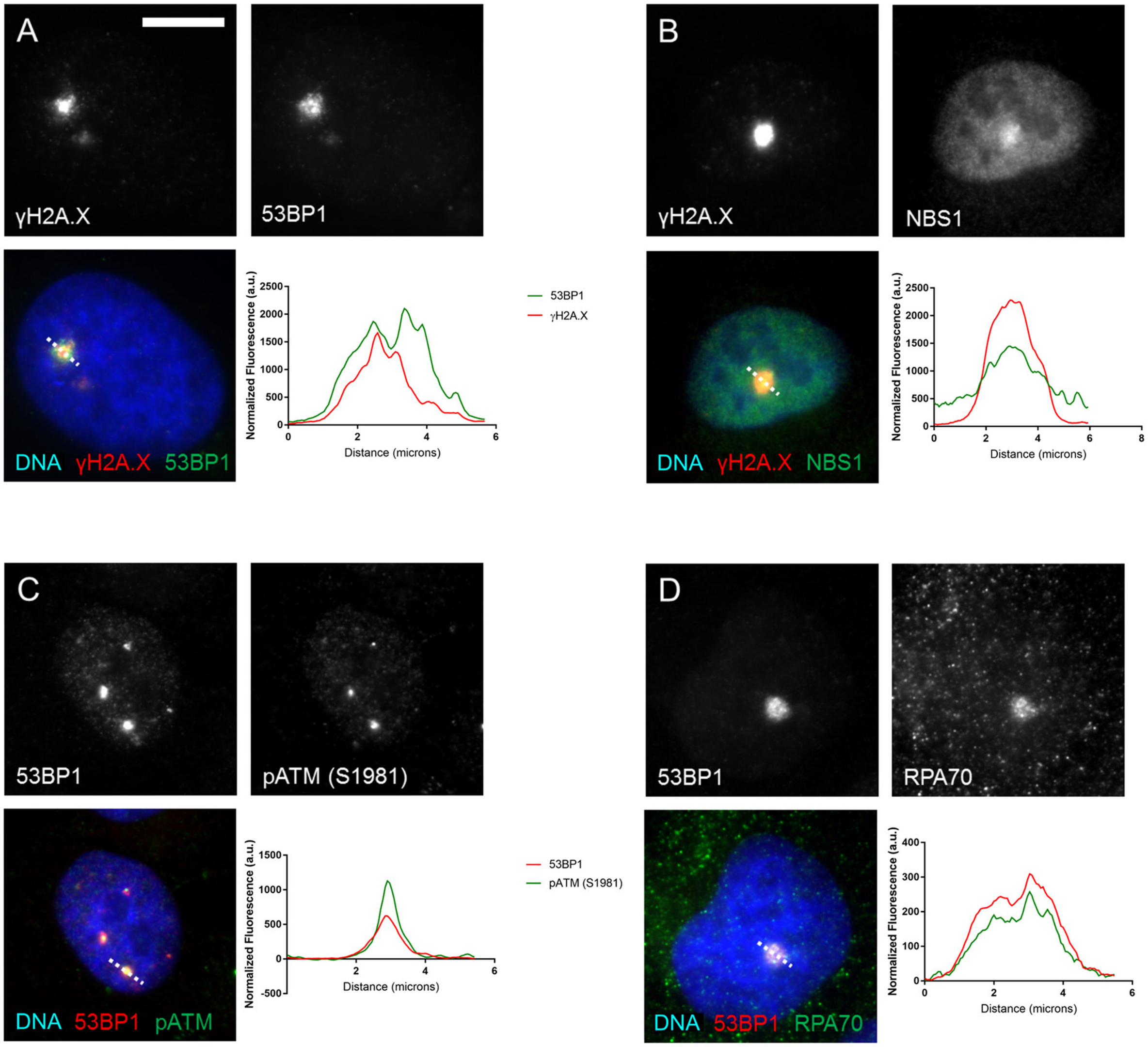

Supplementary Figure 3: Double-stranded DNA damage response/repair proteins colocalize with γH2A.X. Cells were treated with 1µM selinexor for 8 hours, fixed and stained. Representative images are displayed with line scans through foci showing fluorescence intensities along the line. **(A)** 53BP1 (green) colocalizes with γH2A.X (red). **(B)** NBS1 (green) colocalizes with γH2A.X (red). **(C)** pATM S1981 (green) colocalizes with 53BP1 (red). **(D)** RPA70 (green) colocalizes with 53BP1 (red). Scale bar in A = 5µm for all panels.

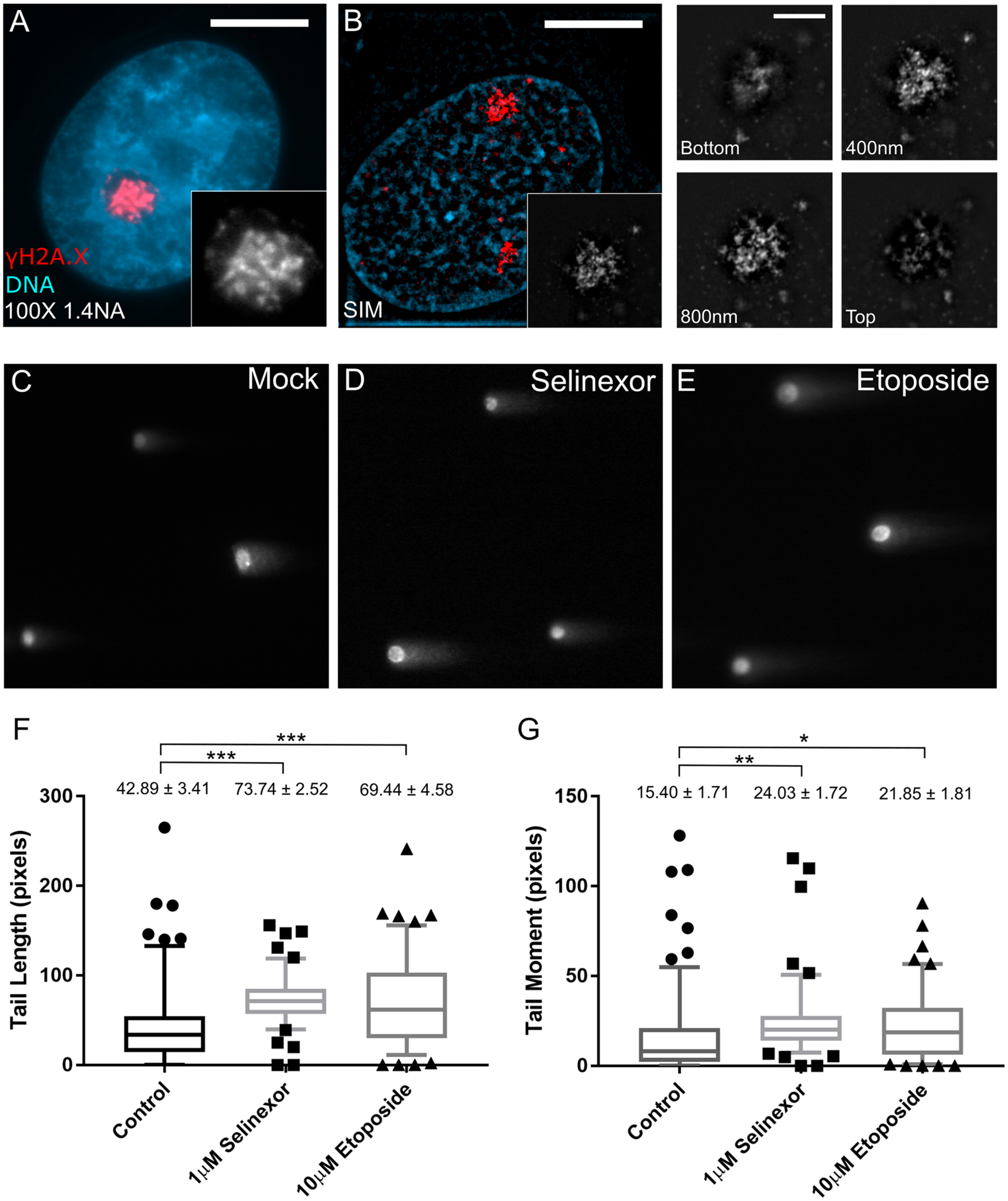

Supplementary Figure 4: High-resolution microscopy and comet assay reveal multiple, clustered DNA double-stranded break sites. HT-1080 cells were treated with 1μM selinexor for 8 hours. **(A)** An example of a cell with DNA damage taken with widefield epifluorescence using a 100X 1.40NA objective. Scale bar = 3μm. **(B)** An example of a cell with DNA damage taken with structured illumination microscopy. Scale bar = 3μm. Four optical Z-sections are shown through the DNA damage structure. Individual foci are resolved within the cluster. Scale bar for Z-sections = 1μm. **(C, D, E)** A comet assay was performed on the cells treated with DMSO (mock), 1μM selinexor or 10μM etoposide for 8 hours. Representative images of comets are shown. **(F)** Quartile analyses of comet tail length. Horizontal line in each population is the median value. The tail length is significantly longer in both selinexor and etoposide treated cells. **(G)** Quartile analyses of the comet tail moment. Horizontal line in each population is the median value. The moment is significantly increased in both the selinexor and etoposide treated populations. Outliers from the 5th and 95th percentile are shown as individual points. Data is from two experiments, total of >100 cells. *** is $p<0.001$, ** is $p<0.01$ and * is $p<0.05$.

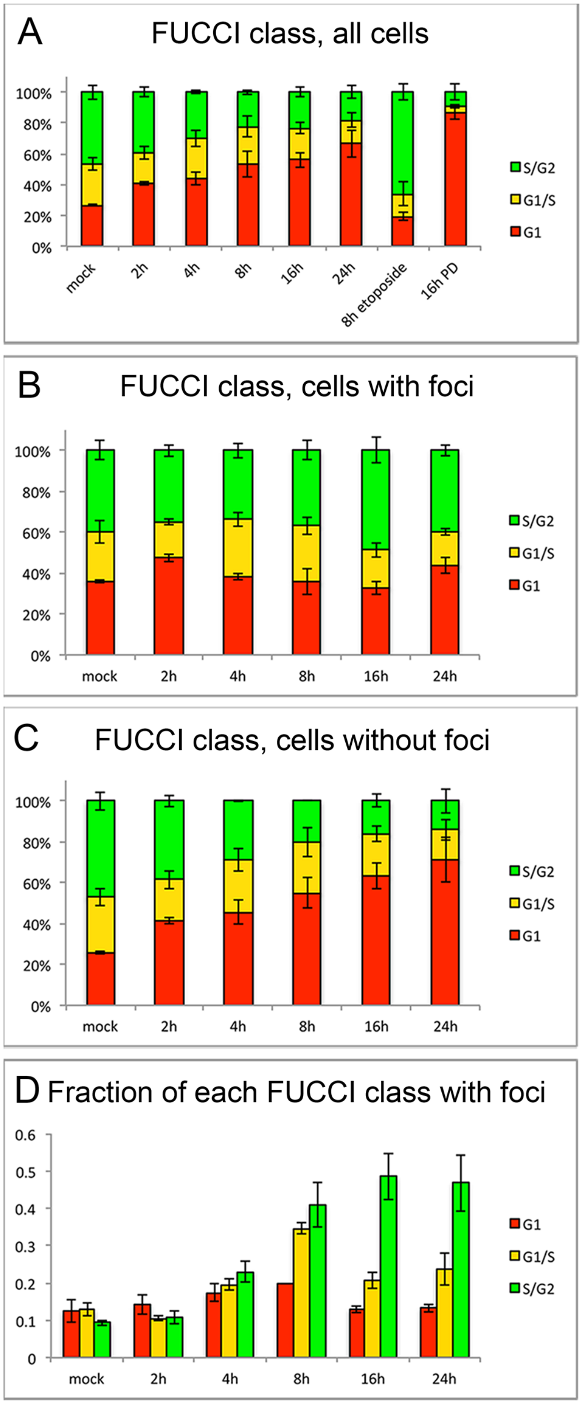

Supplementary Figure 5: Cell cycle effects in HT-1080 correlate with DNA damage status. HT-1080 mKO2-hCdt1(30/120)/mAG-hGem(1/110) cells were treated with 1µM selinexor for increasing amounts of time, and then fixed and immunostained for γH2A.X to detect foci. 10µM etoposide (8 hours, h) and 10µM PD-0332991 (PD, 16h) were included as controls to ensure proper cell cycle response (see Methods). **(A)** The FUCCI class distribution (FUCCI signature) of the entire population of treated cells where cells are in G1-phase (red), G1/S-phase (yellow) or S/G2-phase (green). There is a steady accumulation of G1-phase cells to approximately 70% by 24h. **(B)** Cells with detectable damage foci were analyzed for FUCCI signature. Approximately 60% of cells with foci at early timepoints are present in G1- or G1/S-phase. **(C)** Those cells without detectable damage were analyzed for FUCCI signature. This population of cells shows a signature distinct from cells with damage, and steadily accumulates to approximately 70% G1-phase by by 24h. **(D)** The cell population at each time point was analyzed for the fraction of each FUCCI class that show foci. At early timepoints, cells in G1- or G1/S-phase together show more foci, by by 24h S/G2phase cells more often show foci. For more detail see the text. N > 5000 cells for each time point and condition. Error bars are SEM from three experiments.

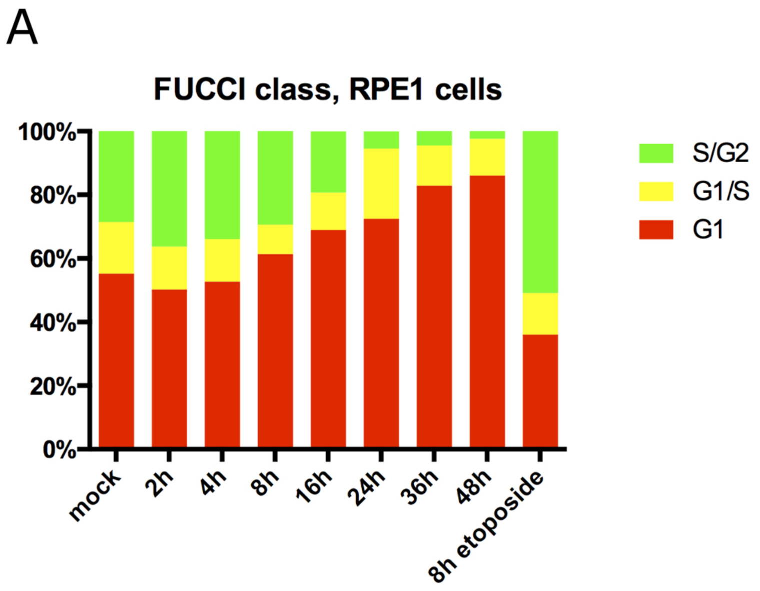

Supplementary Figure 6: Cell cycle effects in RPE1 cells. RPE1 mKO2-hCdt1(30/120)/mAG-hGem(1/110) FUCCI cells were treated with 1µM selinexor for increasing amounts of time and the FUCCI classes were quantified. RPE1 cells do not form significant DNA damage foci after treatment with selinexor (Supplementary Figure K versus L). Over time, the population shifts toward a G1-phase FUCCI signature. By 48 hours (h), the population is >80% G1-phase. This FUCCI signature is similar to that seen in HT-1080 cells that do not show DNA damage foci (Supplementary Figure 5C).

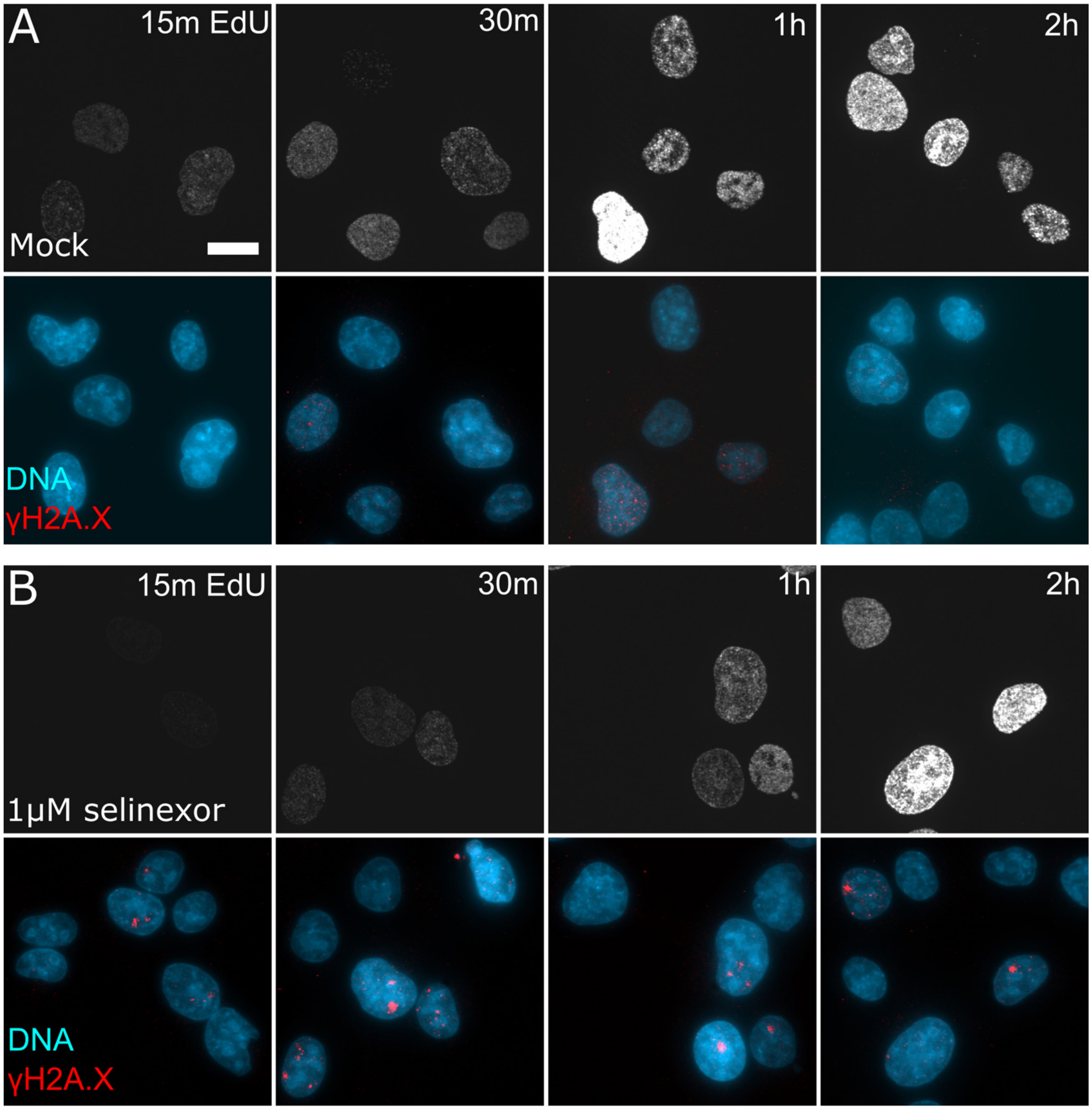

Supplementary Figure 7: Representative images of HT-1080 cells from Figure 4. (A) HT-1080 cells were treated with DMSO (mock) or **(B)** 1μM selinexor for 8 hours (h) and and pulse-labeled with EdU for the last 15, 30, 60, or 120 minutes. Cells were fixed and stained for γH2A.X (red), DNA (blue) and EdU (white). Prominent DNA damage foci are observed in cells that also show some EdU labeling. Scale bar in A = 10μm for all panels.

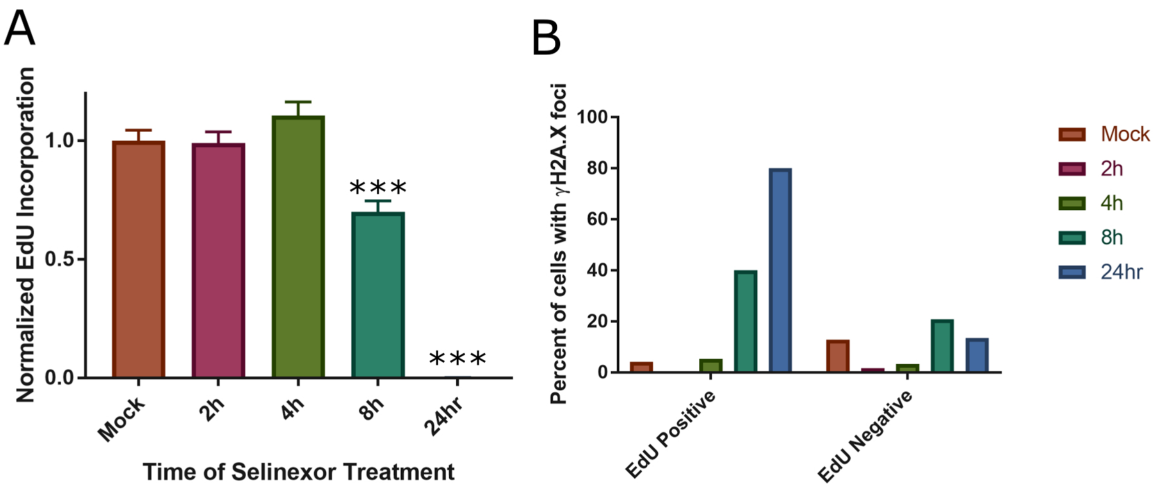

Supplementary Figure 8: Cells with DNA damage foci associate strongly with S-phase cells and S-phase progression defects. U2OS cells were treated with DMSO (mock) or 1μM selinexor for specified times (hours, h). Cells were pulse labeled for for 15 minutes (m) with EdUat the end of treatment. Experimental scheme is as Figure 4A. Cells were then stained for γH2A.X and DNA. **(A)** The mean EdU incorporated in cells is decreased by 8h and negligible by 24h. Error bars are the SEM from >150 cells analyzed per condition. *** is $p<0.001$. **(B)** The population of analyzed cells was divided into two groups, EdU positive and EdU negative. γH2A.X foci were identified and the percentage of cells in each group with foci was quantified. EdU positive cells show damage foci more frequently than EdU negative cells.

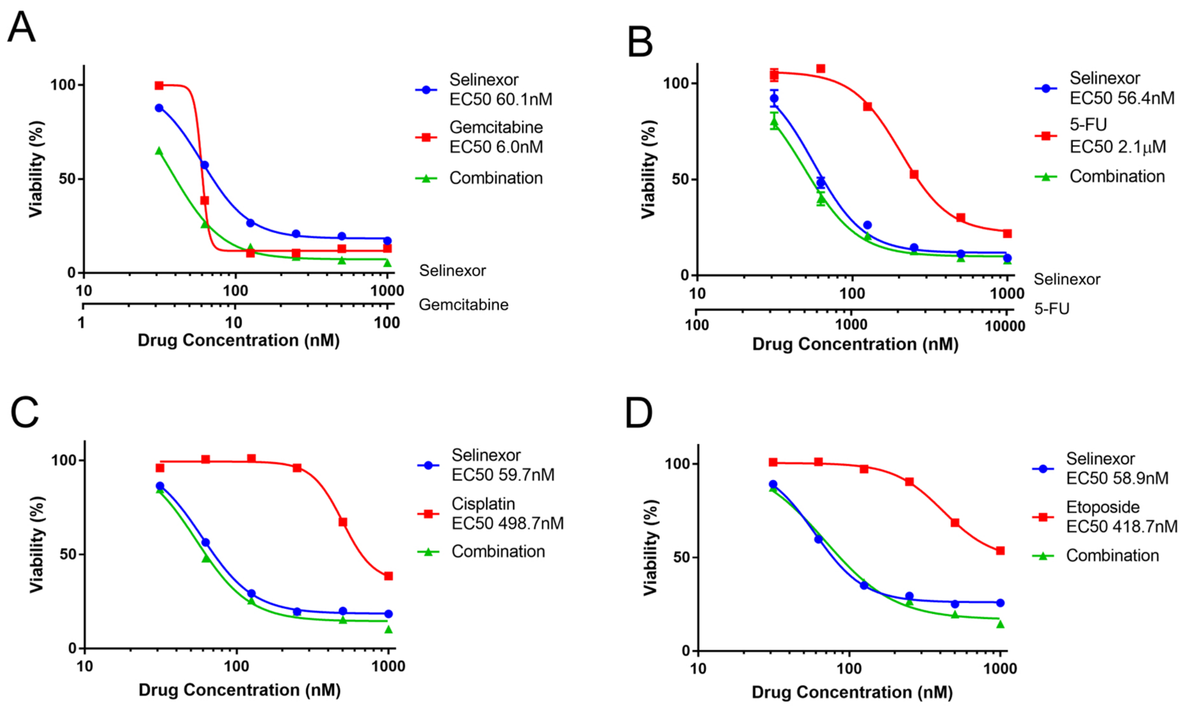

Supplementary Figure 9: Single agent and combination dose responses of selinexor and different classes of DNA damage agents. (A-D) After 72 hours, ATP was measured as a surrogate for cell survival. Survival curves were performed for single agents alone and in combinations with selinexor. EC_{50} concentrations are noted in the respective figure key. Combination indices were calculated in Figure 6 for each equimolar combination dose.

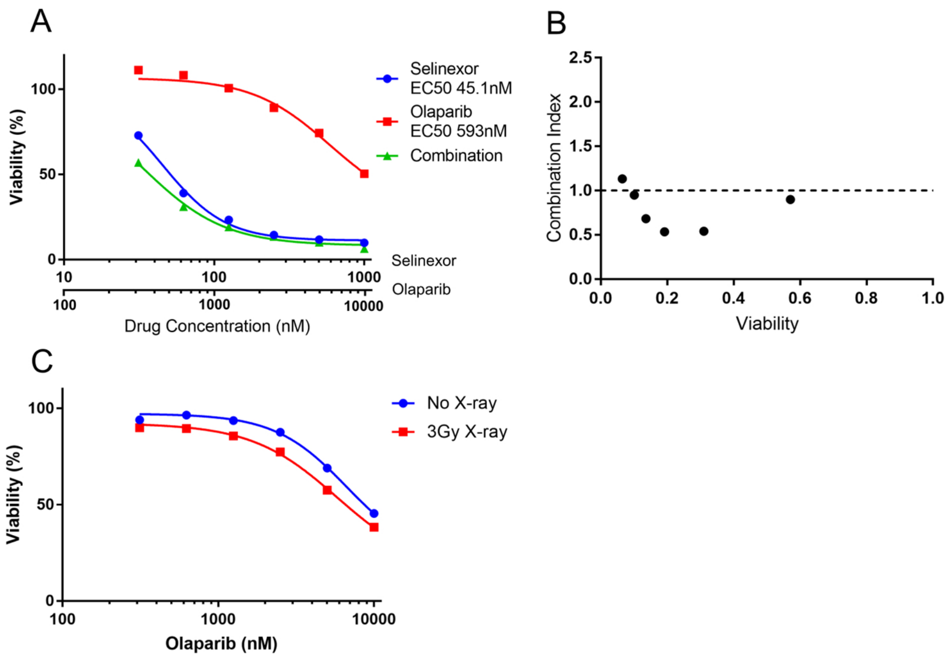

Supplementary Figure 10: Selinexor combines synergistically with the PARP1 inhibitor olaparib. **(A)** Dose-response curves were performed for selinexor and olaparib alone and in combination. After 72 hours, ATP was measured as a surrogate for cell survival. EC_{50} concentrations are noted in the figure key. **(B)** Combination indices for each combination were calculated using the median effect model. The dotted line represents a combination index of 1. Points less than 1 are synergistic whereas points greater than 1 are antagonistic. **(C)** As a control, olaparib was combined with 3Gy of x-irradiation. After 72 hours, ATP was measured as a surrogate for cell survival and apparent combination effects are observed across the olaparib concentration range.

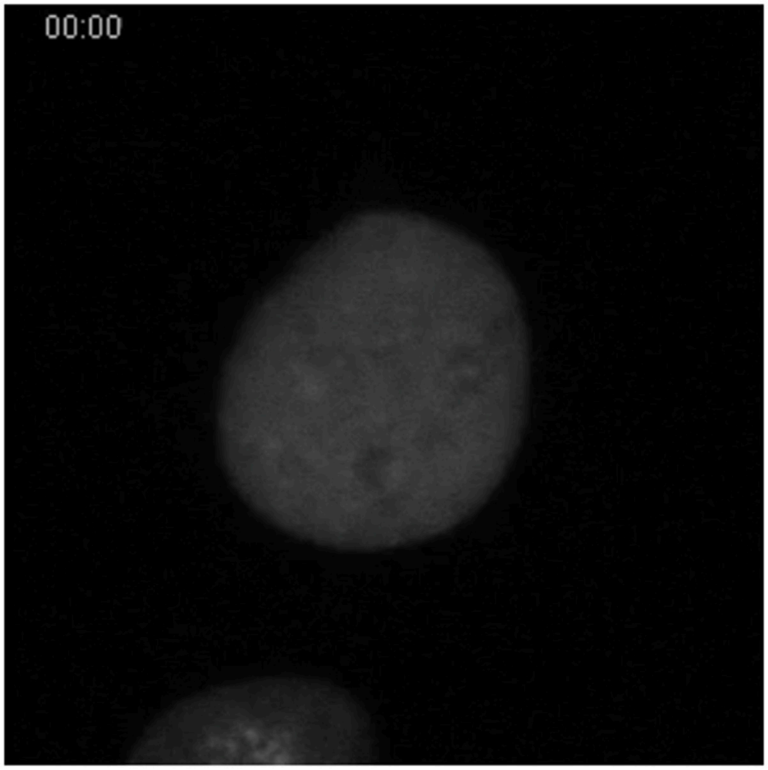

Supplementary Video 1: HT-1080 mCherry-BP1-2 cells treated with 10µM etoposide rapidly acquire DNA damage upon exit from mitosis. These numerous foci appear as those found in γH2A.X stained in fixed cells.

See Supplementary Video 1

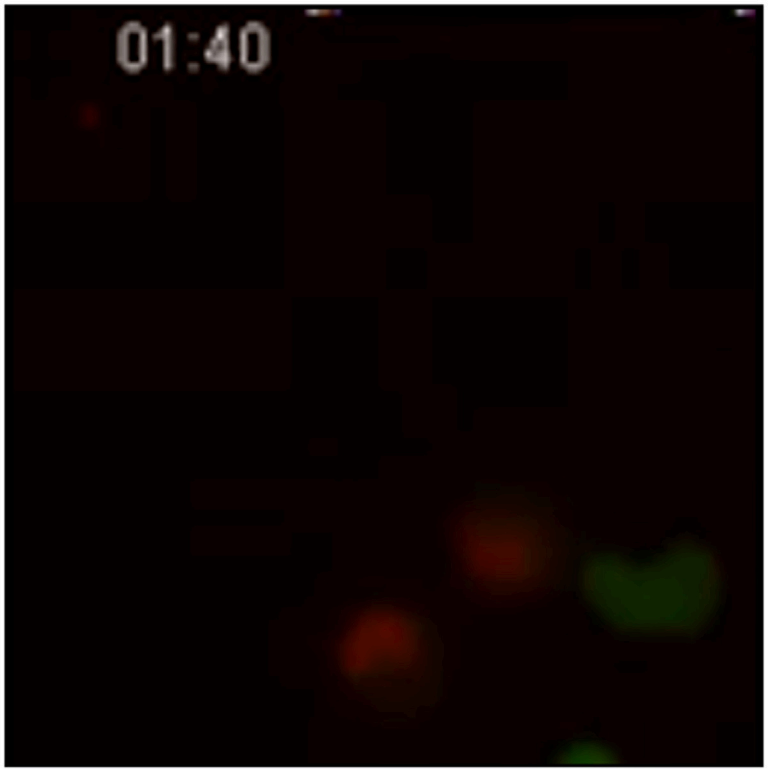

Supplementary Video 2: HT-1080 mAG-hGem(1/110)/mCherry-BP1-2 cell treated with 1μM selinexor acquires DNA damage in G1-phase, progresses to S/G2-phase and then dies. Corresponds with Figure 5A.

See Supplementary Video 2

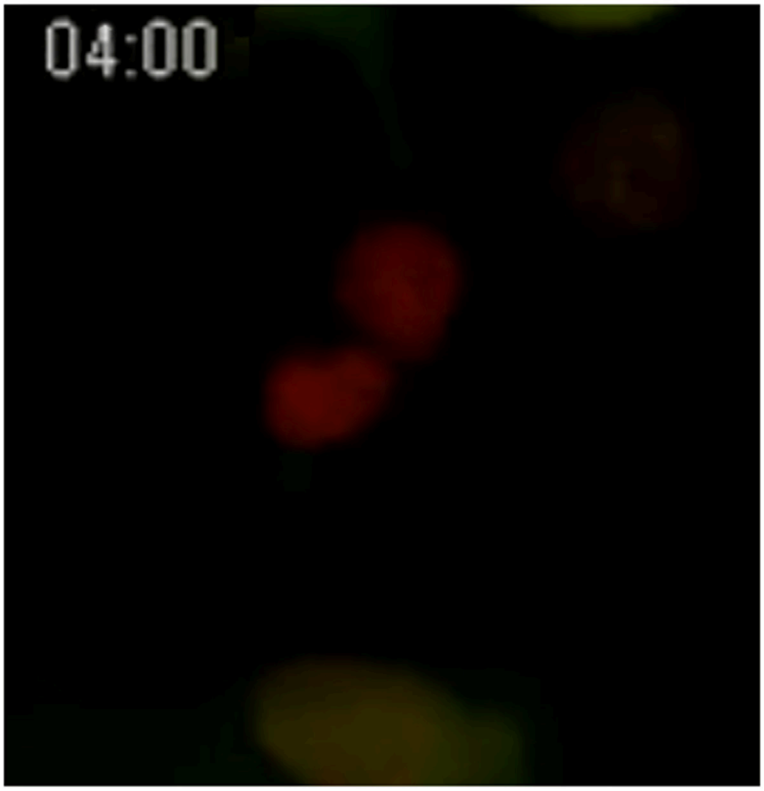

Supplementary Video 3: HT-1080 mAG-hGem(1/110)/mCherry-BP1-2 cell treated with 1μM selinexor acquires DNA damage in G1-phase and remains in a prolonged G1-phase before undergoing death.

See Supplementary Video 3

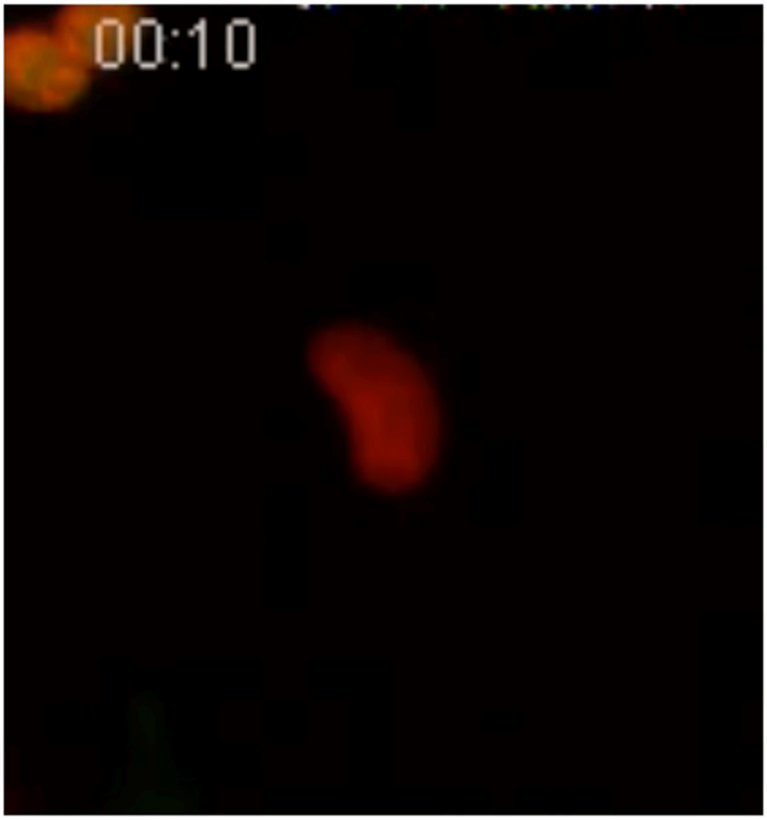

Supplementary Video 4: HT-1080 mAG-hGem(1/110)/mCherry-BP1-2 cell treated with 1µM selinexor progresses from G1-phase to S/G2-phase, acquires damage in S/G2-phase and progresses to cell division. Corresponds with Figure 5B.

See Supplementary Video 4

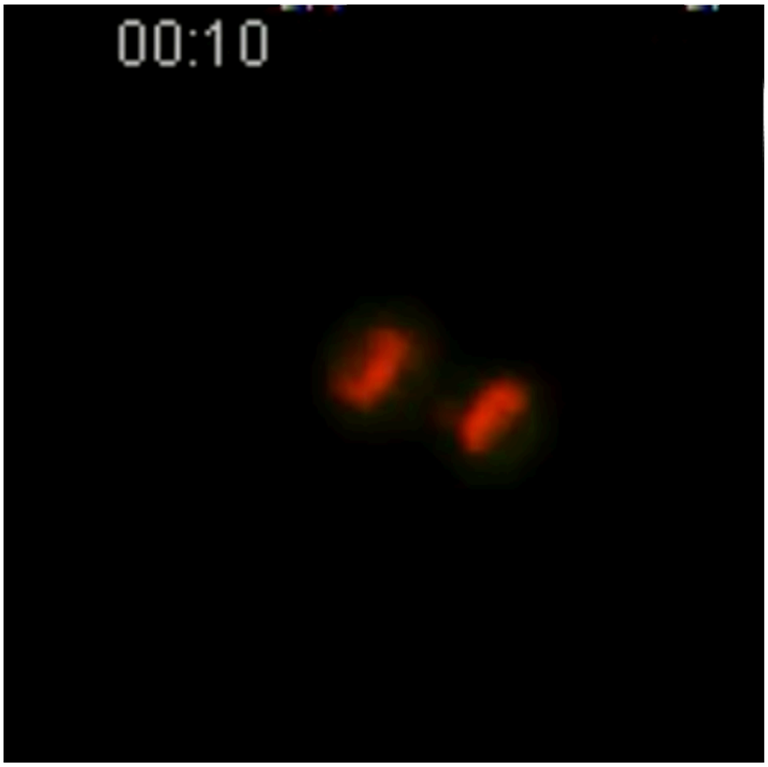

Supplementary Video 5: HT-1080 mAG-hGem(1/110)/mCherry-BP1-2 cell treated with 1µM selinexor acquires DNA damage in G1-phase, appears to repair the damage while progressing through S/G2-phase, and progress to cell division.

See Supplementary Video 5

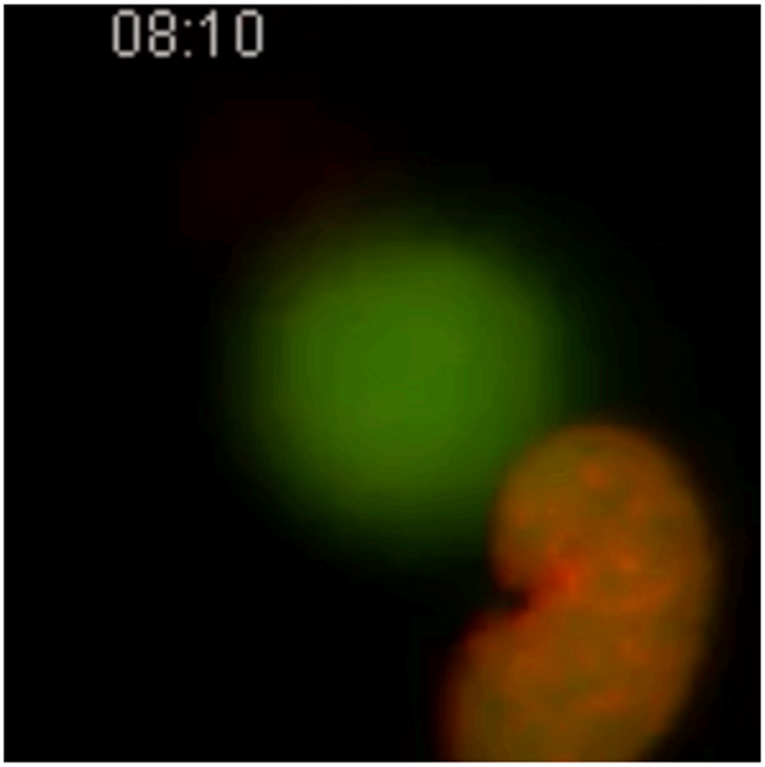

Supplementary Video 6: HT-1080 mAG-hGem(1/110)/mCherry-BP1-2 cell treated with 1µM selinexor acquires DNA damage in G1-phase, repairs the damage, and remains in G1-phase for the duration of the experiment.

See Supplementary Video 6

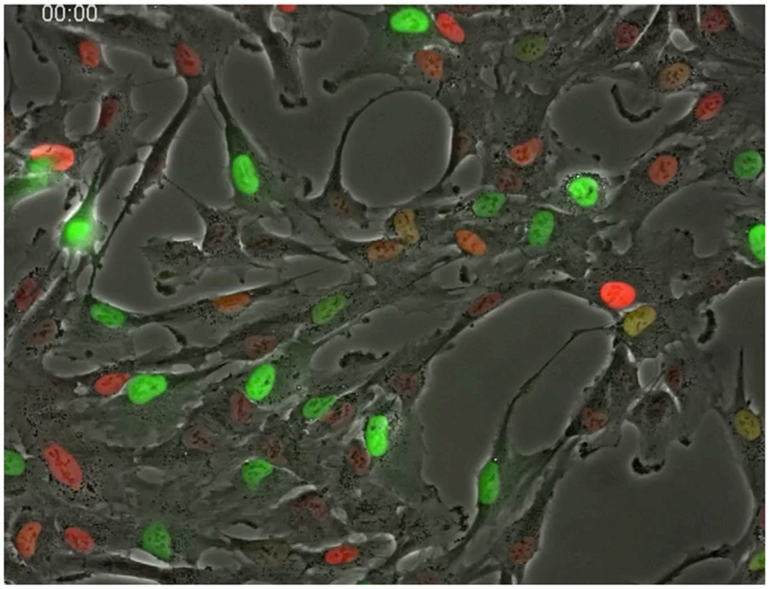

Supplementary Video 7: RPE1 mKO2-hCdt1(30-120)/mAG-hGem(1/110) FUCCI cells treated with 1µM selinexor show strong cell cycle arrest and little cell death. Most cells arrest in a G1-phase stat. Of note: RPE1 cells show no significant DNA damage over mock-treated cells (Supplementary Figure 1) and a FUCCI distribution over time similar to HT-1080 cells that do not form damage (Supplementary Figure 6).

See Supplementary Video 7
